# Red Blood Cells in Platelet-Rich Plasma: Avoid If at All Possible

**DOI:** 10.3390/biomedicines11092425

**Published:** 2023-08-30

**Authors:** Ashim Gupta, Nicola Maffulli, Vijay Kumar Jain

**Affiliations:** 1 Regenerative Orthopaedics, Noida 201301, India; 2 Future Biologics, Lawrenceville, GA 30043, USA; 3 BioIntegrate, Lawrenceville, GA 30043, USA; 4 South Texas Orthopaedic Research Institute (STORI Inc.), Laredo, TX 78045, USA; 5 Department of Musculoskeletal Disorders, School of Medicine and Surgery, University of Salerno, 84084 Fisciano, Italy; n.maffulli@qmul.ac.uk; 6 San Giovanni di Dio e Ruggi D’Aragona Hospital “Clinica Ortopedica” Department, Hospital of Salerno, 84124 Salerno, Italy; 7 Barts and the London School of Medicine and Dentistry, Centre for Sports and Exercise Medicine, Queen Mary University of London, London E1 4DG, UK; 8 School of Pharmacy and Bioengineering, Keele University School of Medicine, Stoke on Trent ST5 5BG, UK; 9 Department of Orthopaedic Surgery, Atal Bihari Vajpayee Institute of Medical Sciences, Dr. Ram Manohar Lohia Hospital, New Delhi 110001, India; drvijayortho@gmail.com

The last decade has seen a noticeable upsurge in the use of biologics, including platelet-rich plasma (PRP), for applications in musculoskeletal regenerative medicine. Clinical trials, case series, systematic reviews and meta-analyses have shown the safety and efficacy of PRP for the management of several musculoskeletal disorders [1]. Additionally, pre-clinical studies have reported the disease-improving capabilities of PRP [1]. Nonetheless, discrepancies in the outcomes for patients and new perceptions have defied the reality of its clinical usage [2]. Specifically, different PRP preparation protocols, using either manual or easily accessible commercial PRP and PRP-like systems, result in formulations with variable compositions, i.e., different amounts of platelets, red blood cells (RBCs) and leukocytes [3]. Moreover, the limited literature and lack of consensus on the standardization of a PRP formulation protocol for different clinical indications contributes to contrasting reported results [3]. Here, we focus on the presence of RBCs in PRP preparations; RBCs represent one of the attributes associated with the efficacy of PRP and are a major discriminant for distinguishing PRP formulations.

RBCs are anuclear, comprise protein-bound heme, and transport oxygen (O_2_) to tissues and carbon dioxide (CO_2_) from tissues to the lungs [4]. The heme and iron within RBCs aid in the binding of O_2_ and CO_2_. The typical life cycle of an RBC is 120 days, and RBC senescence is responsible for their removal from circulation by macrophages. The shear forces involved in whole-blood phlebotomy procedures or imperfect PRP concentration practices can cause damage to RBCs in PRP formulations. This results in the disintegration of the RBC membrane and the secretion of noxious hemoglobin, appraised as plasma-free hemoglobin, iron and hemin [5]. The released molecules can lead to the activation of inflammatory pathways and oxidative stress which, in turn, can cause microcirculatory disfunction, vascular damage and substantial tissue injury, exerting a major cytotoxic effect [4]. Specifically, the accumulation of iron from RBCs results in the formation of destructive oxygen metabolites, inducing the apoptosis of chondrocytes, followed by synoviocyte hypertrophy and the infiltration of the synovial membrane by lymphocytes, leading to degenerative joint damage [6]. This in accordance with a study which evaluated the effect of RBC concentrates on fibroblast-like synoviocytes (FLS) and reported significant cell death and the production of pro-inflammatory mediators [7]. Moreover, exposure to RBCs leads to chondrocyte apoptosis after 4 days of exposure independent of synovial inflammation, which can adversely affect the turnover of the cartilage matrix, including an inability to restore proteoglycan synthesis [8]. This study confirmed the findings of an investigation which evaluated the joint damage induced by blood in a canine model and reported changes in the activity of chondrocytes and the solidarity of the cartilage matrix which, in the long term, can result in joint degeneration [9]. 

Additionally, the administration of RBCs with PRP leads to eryptosis, prompting the secretion of a macrophage migration inhibitory factor (MMIF), a potent cytokine [10]. MMIF can inhibit the migration of macrophages and monocytes and wields intense pro-inflammatory signals to adjacent tissues, thereby hindering the proliferation of fibroblasts and the migration of stem cells and likely causing cellular dysfunction [4]. 

In addition, the function of RBCs in tissue regeneration is yet to be determined. There are no pre-clinical or clinical investigations assessing the efficacy of RBC-rich PRP compared to RBC-poor PRP. Thus, until the role of RBCs is established and the pros of including RBCs in a PRP formulation outweighs its cons, it is essential to reduce or eliminate the content of RBCs in PRP formulations to avoid the damaging effects of hemolysis and eryptosis on musculoskeletal tissues. 
